# Elucidating the Structure-Activity Relationships of the Vasorelaxation and Antioxidation Properties of Thionicotinic Acid Derivatives

**DOI:** 10.3390/molecules15010198

**Published:** 2010-01-06

**Authors:** Supaluk Prachayasittikul, Orapin Wongsawatkul, Apilak Worachartcheewan, Chanin Nantasenamat, Somsak Ruchirawat, Virapong Prachayasittikul

**Affiliations:** 1 Department of Chemistry, Faculty of Science, Srinakharinwirot University, Bangkok 10110, Thailand; 2 Department of Pharmacology, Faculty of Medicine, Srinakharinwirot University, Bangkok 10110, Thailand; 3 Department of Clinical Microbiology, Faculty of Medical Technology, Mahidol University, Bangkok 10700, Thailand; 4 Chulabhorn Research Institute and Chulabhorn Graduate Institute, Bangkok 10210, Thailand

**Keywords:** 1-adamantylthionicotinic acid and derivatives, vasorelaxants, antioxidants, nitric oxide, prostacyclin, molecular modeling

## Abstract

Nicotinic acid, known as vitamin B_3_, is an effective lipid lowering drug and intense cutaneous vasodilator. This study reports the effect of 2-(1-adamantylthio)nicotinic acid (**6**) and its amide **7** and nitrile analog **8** on phenylephrine-induced contraction of rat thoracic aorta as well as antioxidative activity. It was found that the tested thionicotinic acid analogs **6**-**8** exerted maximal vasorelaxation in a dose-dependent manner, but their effects were less than acetylcholine (ACh)-induced nitric oxide (NO) vasorelaxation. The vasorelaxations were reduced, apparently, in both *N*^G^-nitro-L-arginine methyl ester (L-NAME) and indomethacin (INDO). Synergistic effects were observed in the presence of L-NAME plus INDO, leading to loss of vasorelaxation of both the ACh and the tested nicotinic acids. Complete loss of the vasorelaxation was noted under removal of endothelial cells. This infers that the vasorelaxations are mediated partially by endothelium-induced NO and prostacyclin. The thionicotinic acid analogs all exhibited antioxidant properties in both 2,2-diphenyl-1-picrylhydrazyl (DPPH) and superoxide dismutase (SOD) assays. Significantly, the thionicotinic acid **6** is the most potent vasorelaxant with ED_50_ of 21.3 nM and is the most potent antioxidant (as discerned from DPPH assay). Molecular modeling was also used to provide mechanistic insights into the vasorelaxant and antioxidative activities. The findings reveal that the thionicotinic acid analogs are a novel class of vasorelaxant and antioxidant compounds which have potential to be further developed as promising therapeutics.

## 1. Introduction

Nicotinic acid (niacin), also known as vitamin B_3_, has been used as a vitamin supplement in human and animal nutrition [1]. Deficiencies of vitamin B_3_ result in avitaminoses, which lead to skin diseases and nervous disorders [1]. Nicotinic acid is an effective lipid lowering drug and an intense cutaneous vasodilator which causes flushing [2,3]. The flushing has been shown to be mediated by nicotinic acid or orphan receptor (GPR109A or HM74A or PUMA-G) and to involve the formation of vasodilatory prostanoids [4]. Nicotinamide is a derivative of nicotinic acid, and both the amide and acid occur naturally, almost exclusively in bound form [1]. Nicotinamide is a known vasodilator [5] which relaxes smooth muscle by inhibiting myosin light chain kinase [6]. Nicotinic acid has been used as topical vasodilator or skin cream prior to luminous laser treatment [7]. Both nicotinic acid and nicotinamide were also used as cosmetic compositions such as lipstick for providing improve skin feel [8]. 

**Figure 1:**
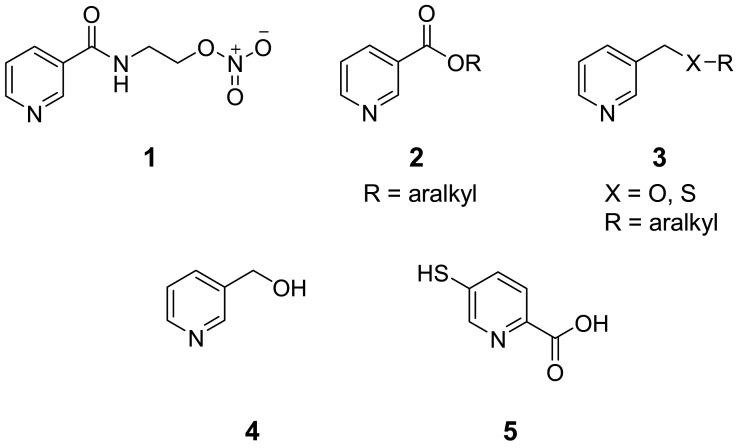
Chemical structure of nicotinic acid derivatives and related compounds **1**-**5**.

Nicotinic acid was introduced into clinical therapy as the first lipid modifying drug [9]. It has been used for decades as an effective antidyslipidaemic drug due to its ability to strongly increase the plasma HDL cholesterol concentration, but decreasing both LDL and total cholesterol including lowering concentration of very low density lipoprotein (VLDL) and plasma triglyceride. Thus, it is referred to as a broad spectrum lipid drug [9]. Such events are important therapeutic measures to reduce cardiovascular morbidity and mortality [9]. It has been shown that the nicotinic acid reduces cardiovascular events in patients with dyslipidemia [2]. However, its clinical use is hindered by harmless, but unpleasant side effect, in particular, a strong flussing [2,3]. In recent years, pharmacological potential of nicotinic acid has drawn considerable interest [4]. Derivatives of nicotinic acid and related compounds e.g. nicorandil (**1**) [10], nicotinate esters **2**, *β*-picolyl ethers (and thioethers) **3** and pyridyl-3-carbinols **4** [11] including nicotinamide adenosine diphosphate (NAD^+^) [12] have been reported as vasodilators. Moreover, picolyl thioethers also exhibited hypotensive activity as compared to ether and carboxyl or amide analogs [11]. It was reported that 5-mercaptopyridine-2-carboxylic acid (**5**, Figure 1) displayed antihypertensive activity [13]. 

**Figure 2:**
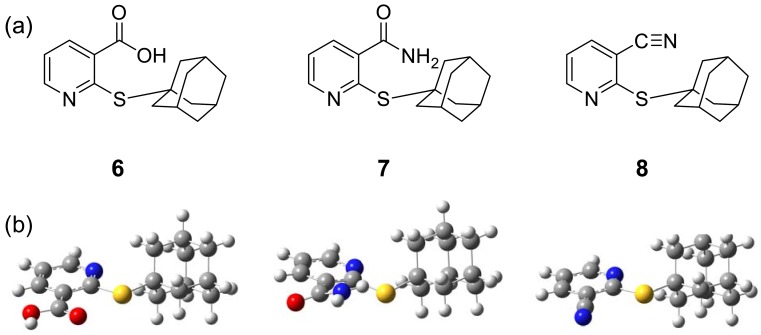
Chemical structures (a) and geometrically optimized structures at B3LYP/6-31g(d) (b) of thionicotinic acid analogs **6**-**8**.

Presently, cardiovascular disease is one of the major health problems accounting for enormous morbidity and mortality worldwide. Therefore, it is crucial to investigate for new vasoactive compounds that affect the functional endothelial cells, which are key regulating cells in the vessel wall. To discover novel vasodilators for medicinal applications, our rational molecular design is based on the documented activity of nicotinic acid derivatives and related compounds. In this context, 1-adamantyl-thionicotinic acid analogs **6**-**8** (Figure 2) are interesting target molecules. The target compounds **6**-**8** were prepared as described previously [14] and evaluated for vasorelaxant and antioxidative activities.

## 2. Results and Discussion

### 2.1. Tested compounds

1-Adamantylthionicotinic acid derivatives **6**-**8** were obtained [14] from the reaction of nicotinic acid, nicotinamide and nicotinonitrile *N*-oxides with 1-adamantylmercaptan in boiling acetic anhydride. The structures of analogs **6**-**8** was confirmed by IR and ^1^H-NMR spectral data and their melting points.

### 2.2. Vasorelaxant activity

Effects of thionicotinic acid analogs **6-8** on vascular function of rat thoracic aorta precontracted with L-phenylephrine (PE) were explored under various conditions; in the presence or absence of inhibitors, namely, *N*^G^-nitro-L-arginine methyl ester (L-NAME) and indomethacin (INDO) and under denuded endothelial cells. In addition, the effects of acetylcholine (ACh) as a positive control, sodium nitroprusside (SNP) as a negative control and vehicle; dimethyl sulfoxide (DMSO) also were studied. Results confirmed that the vasorelaxation of ACh was related to nitric oxide (NO). The DMSO had no effect on induction of vasorelaxations. 

#### 2.2.1. Effect of thionicotinic acid derivatives **6**-**8** on the vascular function of rat thoracic aorta in the presence and absence of L-NAME.

##### Thionicotinic acid **6**

**Figure 3:**
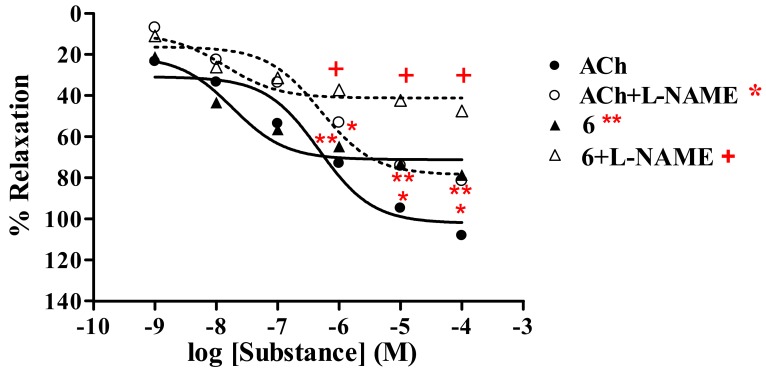
Effect of **6** on the vascular function of rat thoracic aorta in the presence of L-NAME (1 mM) compared with ACh.

The study was performed in the absence and presence of nitric oxide synthase (NOS) inhibitor L-NAME (1 mM). It was found that thionicotinic acid analog **6** exerted vasorelaxation in a dose-dependent manner (Figure 3, Table 1). Maximal vasorelaxation (R_max_) of analog **6** was 78.7%, while ACh produced R_max_ of 108.2%showing ED_50_ of 2.13 × 10^-8^ and 4.72 × 10^-7 M^, respectively. In the presence of L-NAME (1 mM), the dose-response curve of analog **6** was shifted to the right with R_max_ of 47.6% and ED_50_ of 2.5 × 10^-8 M. While ACh displayed R^_max_ of 81.6% with ED_50_ of 4.92 × 10^-7 M. This suggested that thionicotinic acid **6** exhibited vasorelaxant activity by partially producing NO from the endothelial cells.^

##### Thionicotinamide analog **7**

**Figure 4:**
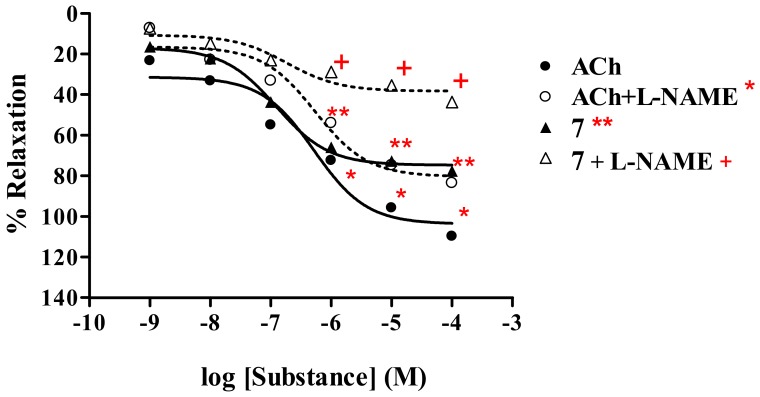
Effect of **7** on the vascular function of rat thoracic aorta in the presence of L-NAME (1 mM) compared with ACh.

Similar results (Table 1, Figure 4) were observed for thionicotinamide derivative **7** and ACh, which exhibited R_max_ of 77.7% and 109.9% with ED_50_ of 1.25 × 10^-7^ and 5.29 × 10^-7 M^, respectively. In the presence of L-NAME (1 mM), the dose-response curve of thionicotinamide **7** and ACh was shifted to the right. R_max_ of derivative **7** and ACh was found to be 43.7% and 83.5%, whereas ED_50_ were 2.66 × 10^-7^ and 5.49 × 10^-7 M, respectively.^

##### Thionicotinonitrile analog **8**

**Figure 5:**
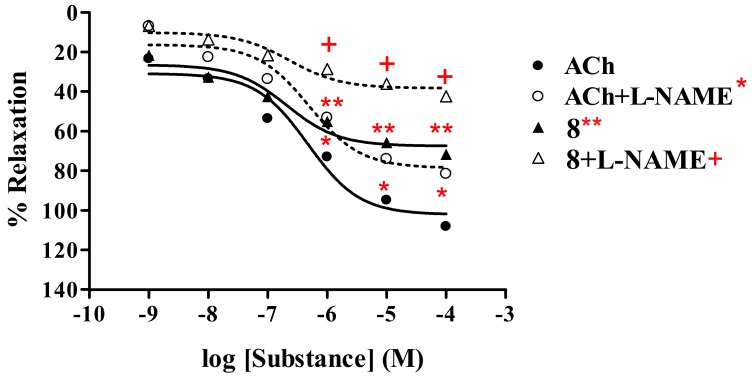
Effect of **8** on the vascular function of rat thoracic aorta in the presence of L-NAME (1 mM) compared with ACh.

Similarly, thionicotinonitrile analog **8** displayed vasorelaxant effect (Table 1, Figure 5) with R_max_ of 71.6% and ED_50_ of 2.44 × 10^-7 M, whereas R^_max_ of ACh was 108.2% with ED_50_ of 4.72 × 10^-7 M^. The dose-response curve of analog **8** was shifted to the right in the presence of L-NAME (1 mM) showing R_max_ and ED_50_ of 42.4% and 3.05 × 10^-7 M, respectively. The R^_max_ of ACh was reduced to 81.6% with ED_50_ of 4.92 × 10^-7 M.^

#### 2.2.2. Effect of endothelial cells on vasorelaxant activity of thionicotinic acid derivatives **6**-**8**

**Figure 6:**
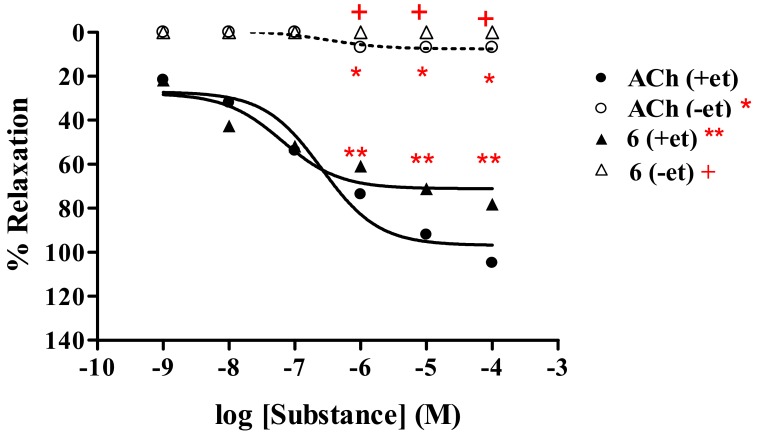
Effect of **6** and ACh on the vascular function of rat thoracic aorta under removal of endothelium (-et) compared with intact endothelium (+et).

The activity was evaluated comparing with Ach with and without intact endothelial cells. The results (Table 2) showed that the vasorelaxation of all tested analogs **6**–**8** was abolished under removal of the endothelial cells. An example of dose response curve such as analog **6** is shown (Figure 6). The similar result was noted for the control; ACh. This confirmed that the vasorelaxant of analogs **6**–**8** was mediated through endothelial producing NO.

#### 2.2.3. Effect of derivatives **6**-**8** on the vascular function of rat thoracic aorta in the presence of cyclo-oxygenase inhibitor (INDO) 

**Figure 7:**
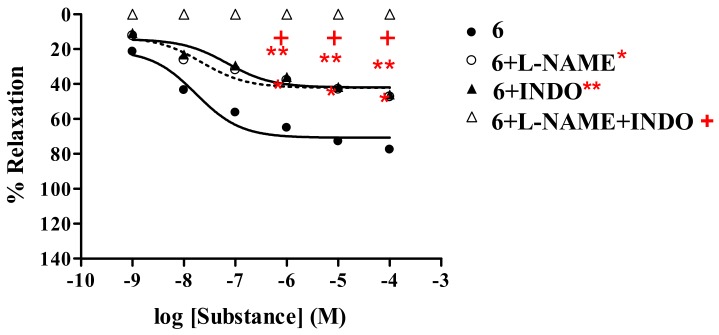
Effect of **6** on the vascular function of rat thoracic aorta in the presence of INDO compared with L-NAME and with L-NAME plus INDO.

The vasorelaxant activity of analogs **6**-**8** was investigated in the presence of INDO (1 mM), compared with L-NAME (1 mM) and L-NAME plus INDO. The results (Table 3) revealed that experiments carried out with L-NAME or INDO showed significant reductions of vasorelaxation (Figure 7) in a dose–dependent manner. 

The inhibitory effect of INDO was comparable to that of L-NAME; as noted from the fact that thionicotinic acid analog **6** showed R_max_ of 47.4% and 46.1%, respectively. However, the antagonistic effects of INDO were stronger than those of L-NAME, as evidenced by the fact that thionicotinamide analog **7** and thionicotinonitrile **8** showed comparable R_max_ of 36.5% and 37.6%, respectively. Moreover, significant reductions of R_max_ were more pronounced in the presence of L-NAME plus INDO. Under such conditions it was found that the vasorelaxation effects of the tested analogs **6-8** and ACh were abolished. However, no significant change was observed by the SNP. The results confirmed that the analogs **6-8** elicited partial vasorelaxation *via* endothelial cells producing NO and prostacyclin (PGI_2_).

The studies have demonstrated that all the tested analogs; 2-(1-adamantylthio)nicotinic acid (**6**), its amide **7** and nitrile **8** derivatives exhibited maximal vasorelaxation in a dose-dependent manner, even though the vasorelaxations are less than those produced by the ACh. Such vasorelaxations are elicited by partial NO production from functional endothelial cells, which is observed by a significant reduction of the activity in the presence of L-NAME (Table 1). The 2-(1-adamantylthio)nicotinic acid (**6**) displayed the highest vasorelaxation with R_max_ of 78.7%, whereas the thionicotinamide (**7**) exhibited comparable R_max_ of 77.7%. The lowest vasorelaxant activity was noted for 2-(1-adamantylthio) nicotinonitrile (**8**) showing R_max_ of 71.6%. Significantly, the thionicotinic acid analog **6** exerts immediate vasorelaxation with ED_50_ of 21.3 nM (Figure 3). This is presumably due to the fact that the nicotinic acid analog **6** has higher affinity for the receptor than the thionicotinamide **7**. Thus, the nicotinic acid **6** is the most potent vasorelaxant. Moreover, the vasorelaxant activities of the tested analogs **6–8** were all abolished by the removal of functional endothelial cells (Table 2, Figure 6). This confirms that the vasorelaxation of analogs **6–8** is modulated *via* NO production by endothelial cells. In fact, ACh involves vasorelaxation by mediating NO, PGI_2_ and endothelium-derived hyperpolarizing factor [15,16,17]. Thus, the experiments were designed and conducted in the presence of cyclooxygenase inhibitor (INDO, 1 mM) compared with L-NAME (1 mM). The results (Table 3) show that the vasorelaxation of the tested compounds (**6–8**) and ACh (Figure 7) is significantly reduced in the dose-dependent manner when compared to that of in the presence of L-NAME. In particular, the antagonistic effects of INDO were stronger than L-NAME for analogs **7** and **8**. Significant reductions of R_max_ were profoundly observed in the presence of L-NAME plus INDO which lead to complete loss of the activity of the tested compounds and ACh. However, there was no significant change of R_max_ produced by the SNP. The data support that the thionicotinic acid and derivatives (**6**-**8**) exhibit vasorelaxation by partial synthesis of NO and PGI_2_ by functional endothelial cells. The former was inhibited by L-NAME, and the latter was inhibited by INDO. It was reported that nicotinic acid itself exerted vasorelaxation *via* mediation of prostaglandin release from vascular function [18,19]. So far, vasorelaxation of the thionicotinic acid analogs **6**-**8** has never been reported in the literature. It is known that NO is an important signaling molecule implicated in cardiovascular function such as vascular tone, whereas PGI_2_ is a powerful vasorelaxants and antioxidant. PGI_2_ is clinically used for treatment of pulmonary hypertension and portopulmonary hypertension [20].

### 2.3. Antioxidative activity

The antioxidative activity of thionicotinic acid derivatives **6-8** was tested using the 2,2-diphenyl-1-picrylhydrazyl (DPPH) and superoxide dismutase (SOD) assays. The results (Table 4) showed that thionicotinic acid **6** was the most potent antioxidant showing 33.20% radical scavenging activity (DPPH) at 333.33 μg/mL, whereas thioamide **7** and thionitrile **8** exerted weak activity (0.57 and 0.30%, respectively). 

The role of NO related to superoxide radical was reported in many studies [21]. Thus, the SOD activity of the thioderivatives **6**-**8** was tested. It was found that the analogs **6**-**8** displayed comparable SOD activity with 15.40–17.31% nitro blue tetrazolium (NBT) inhibition, the thionitrile **8** being the strongest antioxidant.

### 2.4. Molecular modeling of vasorelaxant and antioxidative activities

Previous efforts to elucidate the structure-activity relationship of both vasorelaxant and antioxidative activities were purely based on chemical intuitions on the substituent effects of a series of compounds [22]. In this study, the structure-activity relationship [23,24,25,26,27,28,29] of such activities were discerned in a quantitative manner as provided by molecular descriptors derived from density functional theory (DFT) calculation at the B3LYP/6-31g(d) level.

The energy gap of highest occupied molecular orbital (HOMO) and lowest unoccupied molecular orbital (LUMO) represents an important stability index [30,31,32] for molecules where high HOMO-LUMO gap suggests that the compound is highly stable and is inferred to be relatively inert and less reactive. On the other hand, compounds with low HOMO-LUMO gap are less stable and are therefore more reactive. Such parameter is therefore suitable for assessing the kinetics of vasorelaxant activity as provided by ED_50_. The HOMO-LUMO gap of all the analogs (0.1605-0.1837) was significantly smaller than that of Ach (0.2800) (Table 5). This suggests that the thionicotinic acid analogs are more chemically and kinetically reactive than ACh as the analogs exhibited lower HOMO-LUMO energy gaps than ACh.

One of the commonly used parameters for assessing the relative antioxidative activity is the ionization potential (IP) [33] which essentially measures the electron transferring ability between the antioxidant and radical. IP is typically calculated by taking the difference in energy of a neutral and anionic species of the molecule of interest. To save computational cost, an alternative approach for deriving the IP value can be semi-quantitatively estimated from the negative value of HOMO as proposed by Koopman’s theorem. As the Koopman’s theorem was originally stated to be valid for Hartree-Fock (HF) theory, previous work has shown that a linear correlation exists between the experimental IPs for nine monosubstituted benzene derivatives with both the HF HOMO energies and the DFT HOMO energies [34,35]. Such result supports the use of DFT HOMO energy as a relative measure of IP for the thionicotinic acid analogs.

The DPPH radical scavenging activity of the analogs exhibited a negative correlation of −0.9826 with their estimated IP values (Table 6). The result revealed that **6** was the most potent DPPH radical scavenger with an activity of 33.20% while the least potent DPPH radical scavenger was **8**. Previous efforts had demonstrated that the relative antioxidative activity of a compound can be estimated from quantum chemical calculations [36,37,38,39,40,41]. IP was used as a relative measure of the radical scavenging activities of the thionicotinic acid analogs where low IP value is an indicator of good radical scavenging activity as the antioxidant has a higher probability of losing an electron to scavenge the radical. The experimental results correlated well with the calculated parameter where the best radical scavenger, **6,** also exhibited the lowest IP (0.2184) while the poorest radical scavenger, **8,** also had the highest IP (0.2285).

The SOD activity of the analogs as elucidated by NBT photoreduction provided moderate correlation of 0.6748 with the calculated IPs while strong correlation of 0.8575 was observed with the dipole moments (Table 6). Results for the latter suggest that electron-withdrawing moiety was crucial for the SOD activity as analog **8** possessed the highest dipole moment (4.8092) while exhibiting the greatest NBT photoreduction (17.31% inhibition). The opposite is true for the analogs as electron-donating moeity was found to be crucial for DPPH radical-scavenging activity. In particular, analog **6** with low dipole moment (2.4098) provided good DPPH radical-scavenging activity (33.20%). As dipole moment accounts for the asymmetric distribution of charges in a molecule, therefore analogs with high dipole moment suggests that there exists a higher degree of charge localization. Of the three analogs, **8** possessed the highest dipole moment and also the highest degree of asymmetric charge distribution.

## 3. Conclusions

This investigation discloses novel vasorelaxants and antioxidants represented by 2-(1-adamantylthio)nicotinic acid (**6**) and its amide **7** and nitrile analog **8**. The thionicotinic acid **6** is the most potent vasorelaxant and antioxidant (as discerned from DPPH assay). Significantly, the thionicotinic acid **6** exhibits immediate vasorelaxation with a nanomolar ED_50_. The vasorelaxants are mediated *via* endothelium producing NO and PGI_2_. Molecular modeling analysis revealed that dipole moment is a useful molecular descriptor for assessing the vasorelaxant and antioxidative activities. Vasorelaxant ED_50_ was demonstrated to be well correlated with the calculated HOMO-LUMO energy gap and that the IP was a useful theoretical parameter for assessing the antioxidative activities. The findings show potential development of such thionicotinic acid as promising therapeutics.

## 4. Experimental

### 4.1. General

Melting points were determined on an Electrothermal melting point apparatus (Electrothermal 9100) and are uncorrected. ^1^H-NMR spectra were recorded on a Bruker AM 400 instrument with a 400/100 MHz operating frequency using CDCl_3_ or DMSO-d_6_ solution with tetramethylsilane as an internal standard. Infrared spectra (IR) were obtained on a Perkin Elmer System 2000 FTIR. Column chromatography was carried out using silica gel 60 (0.063–0.200 mm). Thin layer chromatography (TLC) was performed on silica gel 60 PF_254_ (cat. No. 7747 E., Merck). Solvents were distilled prior to use. Chemicals for the synthesis were of analytical grade. Reagents for assays were as follows: PE hydrochloride, SNP, L-NAME, ACh, ketamine hydrochloride, INDO, *α*-tocopherol, DPPH and bovine erythrocyte SOD were obtained from Sigma Chemical Co. (USA). DMSO was purchased from Fluka. Tested compounds were dissolved in DMSO, then the solutions were further diluted with normal saline.

### 4.2. Tested compounds ***6***-***8***

The tested compounds were 2-(1-adamantylthio)nicotinic acid (**6**), 2-(1-adamantylthio)- nicotinamide (**7**) and 2-(1-adamantylthio)nicotinonitrile (**8**). They were prepared as previously described [14] and characterized as follows: compound **6:** ^1^H-NMR (DMSO-d_6_): δ2.22-1.73 (m, 15H, 1-Adm), 7.18 (dd, 1H, *J* = 7.78, 4.82 Hz, H-5), 8.06 (d, 1H, *J* = 7.78 Hz, H-4), 8.56 (d, 1H, *J* = 4.82 Hz, H-6); compound **7:** ^1^H-NMR (CDCl_3_): δ2.13-1.70 (m, 15H, 1-Adm), 6.60 (br, CONH_2_), 7.18 (dd, 1H, *J* = 7.21, 4.72 Hz, H-5), 8.18 (dd, 1H, *J* = 7.21, 1.81 Hz, H-4), 8.53 (dd, 1H, *J* = 4.72, 1.81 Hz, H-6) and compound **8:** ^1^H-NMR (CDCl_3_): δ2.25-1.75 (m, 15H, 1-Adm), 7.08 (dd, 1H, *J* = 7.21, 4.72 Hz, H-5), 7.80 (dd, 1H, *J* = 7.21, 1.81 Hz, H-4), 8.58 (dd, 1H, *J* = 4.72, 1.81 Hz, H-6).

### 4.3. Vasorelaxant assay

#### 4.3.1. Isometric tension measurements

The protocols for handling animals were approved by the Animal Care Committee at the Srinakharinwirot University and done at the National Laboratory Animal Centre, Mahidol University. Male Sprague-Dawley rats (170-250 g) were anesthetized with intraperitoneal ketamine hydrochloride (0.05 mL/kg). The thoracic aorta was quickly removed to cold Kreb-Henseleit buffer containing (mM): 118 NaCl; 4.7 KCl; 1.2 KH_2_PO_4_; 1.2 MgSO_4_·7H_2_O; 11.0 (+)-glucose; 25.0 NaHCO_3_; and 2.5 CaCl_2_·2H_2_O, pH 7.4, aerated with 95% O_2_, 5% CO_2_. After removed debris tissue, the vessel was cut into rings, each 2-3 mm-long and hanged in the organ bath containing Kreb-Henseleit solution at 37 °C, aerated with 95% O_2_, 5% CO_2_ and also connected to a force-displacement transducer (Model MLTO50 Force transducer Range: 50, P.R. China) and equilibrated for 50-60 min under a 1 g resting tension. During an incubation period, the Kreb-Henseleit solution was changed every 20 min. After the incubation period, the maximal contraction of the rings was determined with high dose of PE (10^-5^ M) and then washed 5 times until resting tension was recovered. Isometric tension [42,43] was recorded by Macintosh MacLab 4E AD Instrument connected to computer hard drive. The endothelial intact was examined using high dose of ACh (10^-5^ M) at a level of submaximal tension. If the relaxation response to ACh was less than 80%, the ring would be discarded. Then, the ring was washed 5 times to remove the residue of ACh. The vessel was again equilibrated for 50-60 min and the responses of vessel were conducted by the following protocols. Submaximal contraction was induced using PE (10^-7^ M), then cumulative dose-response curves to the agonists (10^-9^-10^-4^ M). Finally, the dose-response curve of SNP was performed in order to test the functional vessel. With inhibitors (L-NAME or INDO) or vehicle, the vessels were pretreated with such compounds prior to submaximal contraction with PE then determined the endothelial response to the tested compounds. After each cumulative dose-response curve, the thoracic aorta preparation was washed and equilibrated for 50-60 min before employing further dose-response curve of tested compounds.

#### 4.3.2. Statistical analyses

The unpaired two-tailed Student’s t test and one-way ANOVA were used in the statistical analysis when appropriate. *Post-hoc* comparisons of individual groups were conducted using the Tukey-Kramer test. The ED_50_ values for the vasorelaxants were deduced using nonlinear regression analyses (GraphPad Prism 4, GraphPad Software Inc., USA). A *p*-value less than 0.05 was considered significant. The data were expressed as mean ± s.e.m. for the number of animals.

### 4.4. Antioxidative assay

Two assays; DPPH and SOD were used. The antioxidative activity of the tested compounds was elucidated by the DPPH radical scavenging assay [44]. When DPPH (a stable purple color) reacts with an antioxidant, it is reduced to yield a light-yellow colored diphenylpicrylhydrazine. Color changes can be spectrophotometrically measured. In this study, experiment was initiated by preparing 0.2 mM solution of DPPH in methanol. One mL of this solution was added sample solution (1 mg/mL dissolved in methanol, 0.5 mL). After 30 min, absorbance was measured at 517 nm and the percentage of radical scavenging activity was calculated from the following equation:

% Radical scavenging = (1-Abs.sample/Abs.cont) × 100

where Abs.cont is the absorbance of the control reaction and Abs.sample is the absorbance in the presence of sample.

The SOD activity was assayed by measuring inhibition of the photoreduction of NBT [45]. The indirect assay is comprised of several reactions: the photochemically excited riboflavin was first reduced by methionine into a semiquinone, which donated an electron to oxygen to form the superoxide source. The superoxide readily converted NBT into a purple formazan product. In this regard, the SOD activity was inversely related to the amount of formazan formation.

### 4.5. Molecular modeling analysis

The molecular structures of the thionicotinic acid analogs were drawn using GaussView, version 3.09 [46]. Full geometry optimization was performed without symmetry constraints under Gaussian 03W [47] at the density functional theory using the Becke’s three-parameter Lee-Yang-Parr (B3LYP) functional and 6-31g(d) basis set. The following molecular descriptors were derived from the low energy conformer: dipole moment, energies of HOMO and LUMO. The energy gap of HOMO and LUMO was then calculated by taking their difference.

## Figures and Tables

Data represented as mean ± s.e.m. of six experiments, each performed in duplicate. *p < 0.05, ACh versus ACh+L-NAME, **p < 0.05, ACh versus **6**, + p < 0.05, **6** versus **6** + L-NAME.

Data represented as mean ± s.e.m. of five experiments, each performed in duplicate. *p < 0.05, ACh versus ACh+L-NAME, **p < 0.05, ACh versus **7**, + p < 0.05, **7** versus **7** + L-NAME.

Data represented as mean ± s.e.m. of six experiments, each performed in duplicate. *p < 0.05, ACh versus ACh+L-NAME, **p < 0.05, ACh versus **8**, + p < 0.05, **8** versus **8** + L-NAME.

Data represented as mean ± s.e.m. of five experiments, each performed in duplicate. *p < 0.05, ACh (+et) versus ACh (-et), **p < 0.05, ACh (+et) versus **6** (+et), + p < 0.05, **6** (+et) versus **6** (-et).

Data represented as means ± s.e.m. of five experiments, each performed in duplicate. *p < 0.05, **6** versus **6** + L-NAME, **p < 0.05, **6** versus **6** + INDO, + p < 0.05, **6** versus **6** + L-NAME + INDO.

**Table 1:** Vasorelaxant activity of thionicotinic acid analogs **6**-**8**.

Compound	Vasorelaxant activity				
	Without L-NAME			With L-NAME (1mM)	
	R_max_ (%)	ED_50_ (M)		R_max_ (%)	ED_50_ (M)
**6**^a^	78.67 ± 0.48	2.13 × 10^-8^		47.60 ± 0.83	2.50 × 10^-8^
ACh^a^	108.17 ± 1.22	4.72 × 10^-7^		81.59 ± 0.63	4.92 × 10^-7^
**7**^b^	77.69 ± 0.47	1.25 × 10^-7^		43.72 ± 0.70	2.66 × 10^-7^
ACh^b^	109.86 ± 0.65	5.29 × 10^-7^		83.54 ± 0.91	5.49 × 10^-7^
**8**^a^	71.64 ± 0.55	2.44 × 10^-7^		42.36 ± 0.98	3.05 × 10^-7^
ACh^a^	108.17 ± 1.22	4.72 × 10^-7^		81.59 ± 0.27	4.92 × 10^-7^

^a^ Data obtained from six experiments; ^b^ Data obtained from five experiments.

**Table 2:** Effect of endothelial cells on vascular effect of nicotinic acid analogs **6**-**8**.

Compound	Vasorelaxant activity				
	+et ^a^			−et ^b^	
	R_max_ (%)	ED_50_ (M)		R_max_ (%)	ED_50_ (M)
**6**^c^	78.18 ± 0.79	6.64 × 10^-8^		0	−
ACh^c^	104.89 ± 1.33	2.66 × 10^-7^		7.11 ± 0.35	3.13 × 10^-7^
**7**^d^	75.19 ± 0.59	1.05 × 10^-7^		0	−
ACh^d^	103.45 ± 1.12	3.35 × 10^-7^		7.47 ± 0.15	3.13 × 10^-7^
**8**^d^	71.92 ± 0.52	3.82 × 10^-7^		0	−
ACh^d^	103.45 ± 1.12	3.35 × 10^-7^		7.47 ± 0.15	3.13 × 10^-7^

^a^ +et: in the presence of endothelial cells; ^b^ -et: in the absence of endothelial cells; ^c^ Data obtained from five experiments; ^d^ Data obtained from six experiments.

**Table 3:** Effect of inhibitors on vascular effect of nicotinic acid analogs **6**-**8**.

Compound	Vasorelaxant activity							
	−Inhibitor^a^		+L-NAME (1mM)		+INDO (1mM)		+L-NAME (1mM) +INDO (1mM)	
	R_max_ (%)	ED_50_ (M)	R_max_ (%)	ED_50_ (M)	R_max_ (%)	ED_50_ (M)	R_max_ (%)	ED_50_ (M)
ACh^b^	121.7 ± 1.44	9.99 × 10^-7^	81.34 ± 0.77	5.44 × 10^-7^	68.78 ± 0.92	4.58 × 10^-7^	0	−
**6**^b^	77.67±0.66	1.78 × 10^-8^	47.44 ± 0.44	3.55 × 10^-8^	46.05 ± 0.26	7.17 × 10^-8^	0	−
**7**^b^	76.93±0.56	1.23 × 10^-7^	43.22 ± 0.66	3.88 × 10^-7^	36.49 ± 0.60	6.34 × 10^-7^	0	−
**8**^b^	71.47±0.42	2.05 × 10^-7^	42.10 ± 0.65	3.43 × 10^-7^	37.63 ± 0.62	6.14 × 10^-7^	0	−
SNP^c^	120.81±1.18	3.16 × 10^-7^	116.70 ± 1.30	3.17 × 10^-7^	112.93 ± 0.61	3.16 × 10^-7^	104.98±1.41	3.17×10^-7^

^a^ -Inhibitor: in the absence of L-NAME or INDO; ^b^ Data obtained from five experiments; ^c^ Data obtained from six experiments.

**Table 4:** Antioxidative activities of analogs **6**-**8**.

Compound	% DPPH radical scavenging activity^a^	% NBT inhibition^b^
**6**	33.20	15.40
**7**	0.57	15.45
**8**	0.30	17.31

^a^ Concentration of 333.33 μg/mL was used in the assay. *α*-Tocopherol was used as a positive control.

^b^ Concentration of 300 μg/mL was used in the assay. Native SOD (4140 U/mg) from bovine erythrocytes was used as a standard.

**Table 5:** Calculated molecular descriptors derived from B3LYP/6-31g(d) as correlated with the vasorelaxant ED_50_.

Compound	Dipole moment (Debye)	IP (eV)	HOMO-LUMO gap (eV)	ED_50_ (M)
**6**	2.4098	0.2184	0.1605	2.13×10^-8^
**7**	3.6920	0.2265	0.1837	1.25×10^-7^
**8**	4.8092	0.2285	0.1669	2.44×10^-7^
ACh	13.1100	0.3976	0.2800	4.72×10^-7^
Correlation with ED_50_	0.9594	0.9038	0.8879	

**Table 6:** Calculated molecular descriptors derived from B3LYP/6-31g(d) as correlated with the antioxidative activities.

Compound	Dipole moment (Debye)	IP (eV)	HOMO-LUMO gap (eV)	DPPH (%)	NBT (%)
**6**	2.4098	0.2184	0.1605	33.20	15.40
**7**	3.6920	0.2265	0.1837	0.57	15.45
**8**	4.8092	0.2285	0.1669	0.30	17.31
Correlation with DPPH	-0.8885	-0.9826	-0.7086		
Correlation with NBT	0.8575	0.6748	-0.2276		
